# Evidence for a rosiaite-structured high-pressure silica phase and its relation to lamellar amorphization in quartz

**DOI:** 10.1038/s41467-023-36320-7

**Published:** 2023-02-04

**Authors:** Christoph Otzen, Hanns-Peter Liermann, Falko Langenhorst

**Affiliations:** 1 Institute of Geoscience, Friedrich-Schiller-University Jena, Carl-Zeiss-Promenade 10, 07745 Jena, Germany; 2 Deutsches Elektronen-Synchrotron DESY, Notkestr. 85, 22607 Hamburg, Germany; 3 Hawai’i Institute of Geophysics and Planetology, School of Ocean and Earth Science and Technology, University of Hawai’i at Manoa, Honolulu, HI 96822 USA

**Keywords:** Mineralogy, Materials science

## Abstract

When affected by impact, quartz (SiO_2_) undergoes an abrupt transformation to glass lamellae, the planar deformation features (PDFs). This shock effect is the most reliable indicator of impacts and is decisive in identifying catastrophic collisions in the Earth´s record such as the Chicxulub impact. Despite the significance of PDFs, there is still no consensus how they form. Here, we present time-resolved in-situ synchroton X-ray diffraction data of single-crystal quartz rapidly compressed in a dynamic diamond anvil cell. These experiments provide evidence for the transformation of quartz at pressures above 15 GPa to lamellae of a metastable rosiaite (PbSb_2_O_6_)-type high-pressure phase with octahedrally coordinated silicon. This phase collapses during decompression to amorphous lamellae, which closely resemble PDFs in naturally shocked quartz. The identification of rosiaite-structured silica provides thus an explanation for lamellar amorphization of quartz. Furthermore, it suggests that the mixed phase region of the Hugoniot curve may be related to the progressive formation of rosiaite-structured silica.

## Abstract (web summary)

Rapid compression experiments on quartz provide evidence for a metastable high-pressure phase with rosiaite structure. The phase forms as lamellae and breaks down to glass during decompression. These discoveries may solve the enigma of lamellar amorphization of quartz during impact events.

## Introduction

Asteroid and comet impacts played a decisive role in the evolution of the Earth including the formation of the Earth–Moon system^1^, the delivery of today’s water^2^, catastrophic mass extinctions^3^, and potentially the emergence of life^4^. Traces of impact events on Earth are manifested by peculiar structural alterations in minerals, with amorphous lamellae in quartz playing the most fundamental role^5,6^. Quartz is ubiquitous in the continental crust and is thus affected by almost every continental impact. Its lamellar amorphization is a reliable indicator and the most applied diagnostic tool in estimating magnitudes of impacts.

High-pressure phase transformations of quartz, in particular the phenomenon of pressure-induced amorphization, have thus been the subject of intense research for more than half a century, employing ever more sophisticated techniques to probe simulated impacts. While the stable high-pressure polymorphic phases coesite and stishovite are formed only in negligible amounts in shock processes, amorphization is the dominant response of quartz under such dynamic conditions^5,7,8^. This solid-state transformation occurs systematically through the formation of planar deformation features (PDFs)^9^, i.e. amorphous lamellae oriented parallel to rational crystallographic planes. The abundance and thickness of PDFs increase over a pressure interval of 20–35 GPa until the entire quartz is converted into so-called diaplectic glass^5,10^. This pressure interval coincides precisely with the mixed phase regime of the Hugoniot curve, the region of peak shock states where the compressibility of quartz suddenly increases^11–13^. The interpretation of the mixed phase regime with PDF formation, however, is uncertain, because the formation mechanism of PDFs is controversial in itself.

Early formation models assumed that the glass either directly formed during compression or as a reversion product of stishovite, the stable high-pressure polymorph^10–14^. However, additional dynamic and static experiments indicate that transitions to metastable high-pressure phases can occur prior to or during amorphization and thus may be related. In an early diamond anvil cell (DAC) study on quartz, X-ray diffraction peaks of a metastable quartz II phase with an unknown crystal structure were detected at 21 GPa and room temperature^15^. The formation of quartz II was later confirmed at similar pressures, but it disappears at slightly higher pressures of 25–26 GPa and is replaced by a monoclinic P2_1_/c phase^16–18^. In a recent in situ gas gun shock compression study, X-ray diffraction peaks of another metastable phase with defective niccolite (NiAs)-structure were also reported in parallel to amorphization^19^. The disordered distribution of Si cations in this hexagonal base structure can plausibly be explained by suppressed atom diffusion into ordered positions due to the short timescale of shock compression. If a high-pressure transition could, however, occur through a diffusionless displacive mechanism, an ordered Si distribution could be attained immediately. Such a mechanism was proposed to produce the monoclinic P2_1_/c phase^20,21^. A diffusionless mechanism was also proposed in a recent density functional theory (DFT) study resulting in Li_2_ZrF_6_-structured silica under non-hydrostatic compression^22^.

Similar experimental results of both shock and static compression techniques led to the suggestion that DAC experiments are a useful alternative to simulate pressure-induced amorphization of naturally shocked quartz^23,24^, although the timescales of both compression approaches are very different. Pressures are commonly applied within less than 1 µs in shock experiments (i.e., strain rates of 10^7^ to 10^8^ s^−1^)^25^, while conventional DAC experiments are run for minutes to hours. We note, however, that none of these techniques seem to be ideal to simulate large-scale impact events, since the rise time of shock waves in nature is up to milliseconds (i.e., strain rates ≥ 10^2^ s^−1^), due to target and projectile irregularities (lithological heterogeneities, pore collapse, irregular projectile shape, etc.), while pressures last for seconds^26^. Here, we have redesigned the experimental approach to reproduce pressure-induced phase transformations of quartz to metastable crystalline and amorphous states by means of controlled rapid membrane-driven compression experiments in the DAC. The structural changes were simultaneously monitored through time-resolved in situ synchrotron X-ray diffraction. The membrane-driven DACs permitted compression of single-crystal quartz at the timescale of tens of seconds, which was sufficient to obtain good-quality diffraction patterns (for details see Supplementary Information). The experiments were performed under non-hydrostatic conditions at room temperature on oriented quartz discs, covering the pressure interval of the mixed phase regime.

## Results

### Time-resolved detection of phase transformations

**Fig. 1:**
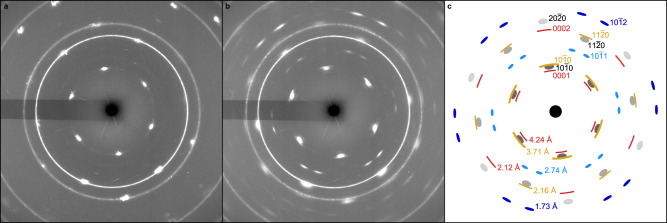
X-ray diffraction patterns of quartz single crystal compressed along the [0001] direction. The single-crystal diffraction patterns (**a**) and (**b**) were collected during compression at 10 and 25 GPa. Circular Debye rings correspond to the polycrystalline gold standard used as a pressure calibrant. (**c**) represents a sketch of the peaks of quartz (gray) and the high-pressure phase (colored) at 26 GPa (cf. (**b**)). The red, orange, and blue reflections refer to different orientations of the same high-pressure phase within the quartz single crystal (cf. Figure 5).

Among the experiments with different orientations of quartz, the uniaxial compression experiments along the [0001] direction — the orientation with the highest symmetry of quartz — provides the most comprehensive understanding of the transformation behavior of quartz (Fig. 1). The compression along the *c*-axis of quartz is certainly not the common situation in natural impacts, but the optimum detection and interpretation of phase transformations occurring in single-crystal quartz require the acquisition of the two-dimensional X-ray diffraction patterns in a symmetrical orientation of the sample. At pressures below 15 GPa, the [0001] single-crystal diffraction patterns represent the trigonal symmetry of quartz through distinct and broadened diffraction peaks of prismatic planes (Fig. 1a). When increasing pressure beyond 15 GPa, additional peaks of a high-pressure phase occurred displaying clear crystallographic relationships to the quartz peaks (Fig. 1b). The interplanar spacings of these additional peaks are 4.24, 3.71, 2.71, 2.12, 1.93, 1.73, and 1.55 Å at 26 GPa. These spacings are very similar to the spacings of 3.66, 2.66, and 1.48 Å reported originally for quartz II and to the additional spacings of 2.0 and 1.87 Å reported in the subsequent study^27^. On the contrary, the peak at 4.24 Å remained unnoticed also in later studies^16–18^ but turns out to be essential for the identification of the crystal structure. During decompression, the new diffraction peaks fade gradually away.

**Fig. 2:**
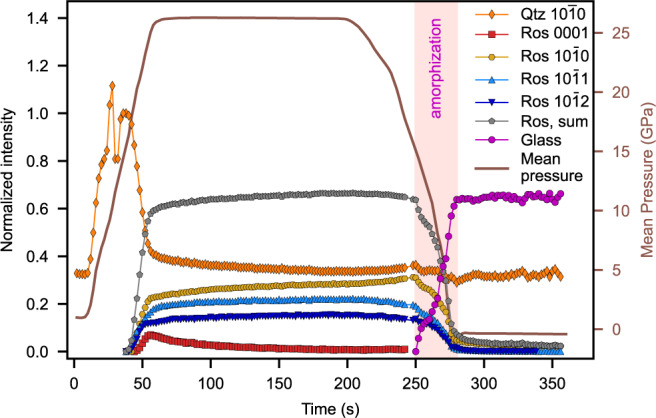
Variation of the normalized X-ray diffraction intensities of the main $10\bar{1}0$ peak of quartz (Qtz) and of the additional diffraction peaks of the high-pressure silica phase (Ros) at the approximate interplanar spacings of 4.2 (0001), 3.7 $(10\bar{1}0)$, 2.7 $(10\bar{1}1)$, and 1.7 Å $(10\bar{1}2)$. The plot additionally shows the normalized sum of peak intensities for the high-pressure phase and the normalized diffuse background intensities, representing the glass fraction.

The X-ray diffraction peak intensities of quartz and the high-pressure phase have been evaluated and normalized to estimate the proportions of the two coexisting phases during compression and decompression (Fig. 2, Supplementary Fig. 1). The absolute intensity of the main 10$\bar{1}$0 quartz peak increases during compression up to 10 GPa and then fluctuates strongly. We observed this behavior also in experiments on other materials and attribute it to the high non-hydrostaticity causing strong crystal lattice distortions in the experiments. At pressures above 15 GPa, the intensity of the main 10$\bar{1}$0 quartz peak decreases significantly, while simultaneously diffraction spots of a high-pressure phase appear. Their intensities increase up to the maximum pressure of the experiment at 26 GPa. The intensities of the new peaks decrease drastically upon decompression, whereas the 10$\bar{1}$0 peak of quartz retains its intensity. Contrary to the structurally similar framework silicate plagioclase, which is reported to recover partially from the amorphous state^28^, we have thus no indication of such a phase memory effect in quartz^29^.

**Fig. 3:**
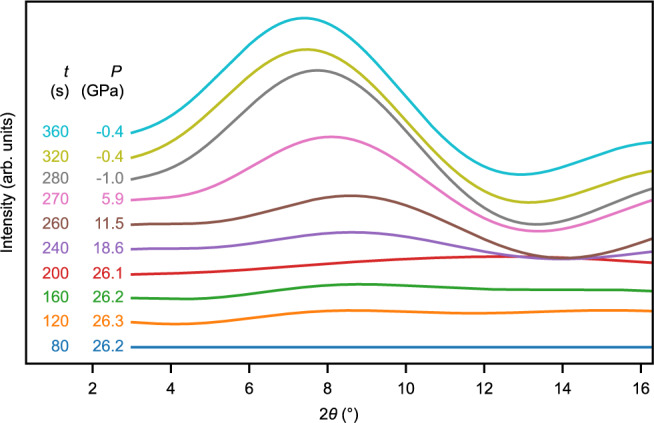
Stacked relative X-ray diffraction intensities of backgrounds for various times (*t*) and pressures (*P*) during loading (80–200 s) and unloading (200–360 s). The intensities display the difference of each background with respect to the reference background at 80 s. It is visible that the diffuse background intensity in the low 2θ region progressively increases due to the formation of amorphous material.

The observation of these intensity variations points to the incipient formation of the high-pressure phase above a pressure of 15 GPa and its subsequent growth upon further compression to 26 GPa at the expense of the quartz. The high-pressure phase gradually vanishes upon decompression from 26 GPa, while an increase in diffuse scattering background emerges as a broad ring in the low 2θ angle region of 7–8°, which is interpreted as the diffuse scattering of amorphous material (Fig. 3). Contemporaneously, the intensity of the 10$\bar{1}$0 quartz peak remains, however, constant until the sample is completely decompressed. The plausible explanation for this behavior is that the high-pressure phase is metastable and amorphizes upon decompression. Our quantitative evaluation of the transformations agrees with the aforementioned earlier study, which regarded a metastable transition of quartz as an intermediate stage towards amorphization^15^.

### Observation of amorphous lamellae in recovered samples

**Fig. 4:**
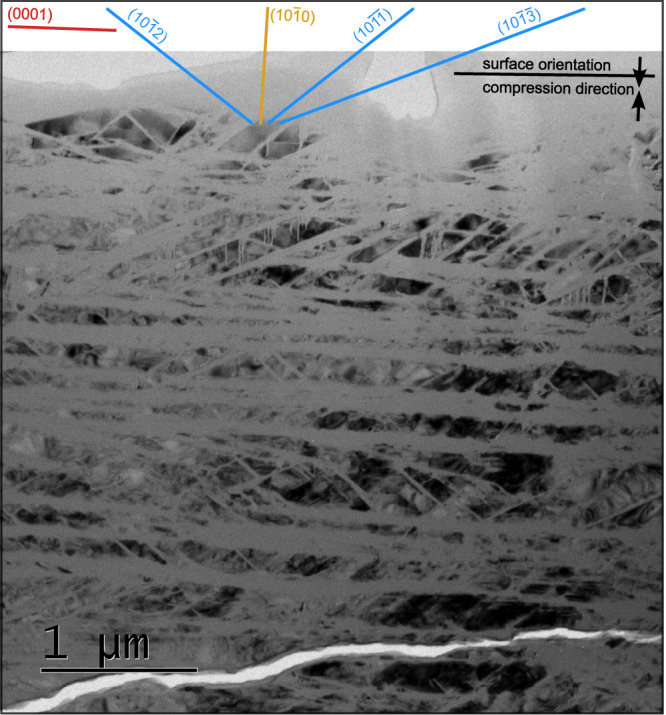
Bright-field transmission electron microscope (TEM) image of recovered quartz disc prepared by focused ion beam preparation. The image shows abundant amorphous lamellae parallel to basal, rhombohedral, and prismatic planes in quartz compressed along the *c*-axis (vertical in the image). The viewing direction is along the [0$\bar{1}$10] direction, i.e. vertical to the compression direction.

The conclusion that the metastable high-pressure phase amorphizes during unloading is corroborated by subsequent transmission electron microscope (TEM) observations of recovered sample discs. Thin foils were extracted perpendicular to the (0001) surface of the single-crystal discs by the focused ion beam technique, providing insights into the subsurface structure of the specimens after the experiment. Bright-field TEM images show the original quartz single crystal to be crosscut by numerous amorphous lamellae with orientations (0001), {10$\bar{1}$2}, {10$\bar{1}$3}, and additional ones that have been described for PDFs in naturally shocked quartz^5,30^ (Fig. 4). Besides diffuse scattering, originating from the amorphous lamellae, the corresponding selected area electron diffraction pattern reveals only diffraction spots that are compatible with the quartz structure. There are no remnant diffraction spots that could be attributed to the metastable high-pressure phase, suggesting its complete breakdown to glass after decompression and removal of the sample disc from the diamond anvil cell. An evaluation of areas occupied by the glass lamellae versus those of crystalline quartz substantiates the X-ray result that about 60% of the sample is amorphous after decompression (Fig. 4). Since the glass formation is correlated with the breakdown of the high-pressure phase (Fig. 2), we conclude that the glass lamellae represent former lamellae of the high-pressure phase.

### Crystal structure of the metastable phase

Questions that remain are: (1) what is the crystal structure of the metastable high-pressure phase and (2) what is its orientation relationship to quartz? To address these questions, the crystallographic meaning of the interplanar spacings of the diffraction peaks attributed to the metastable phase needs to be examined. Almost all crystal structures of high-pressure phases (stishovite, CaCl_2_-type silica, seifertite, etc.) with octahedral coordination of silicon can be simply represented by an idealized hexagonal close-packed oxygen sublattice, in which half of the octahedral sites are filled with silicon in different ordering schemes^20^. The only exception is the defective or modified niccolite (d-NiAs) structure, in which half of the octahedral sites are randomly filled by silicon atoms. This most general description of the structure of high-pressure polymorphs possesses the space group symmetry and dimensions of a perfect hcp lattice (P6_3_/mmc) with *a* = 2.563 Å and *c* = 4.112 Å^31,32^. This phase was reported to occur in diamond anvil cells as well as shock experiments^18,19,31,32^ and it was recently suggested to be the phase that forms in the mixed phase regime of the Hugoniot curve^19^. The defective niccolite phase is historically labeled to be Fe_2_N structured^31,32^. However, this comparison may be misleading because Fe_2_N has two polymorphs (ɛ and ζ) both of which are not hexagonal since the octahedrally coordinated Nitrogen is ordered^22,33^. The ζ phase possesses the orthorhombic seifertite (α-PbO2) structure, while the ɛ phase exhibits the trigonal Li_2_ZrF_6_ structure.

The interplanar spacings determined from our single-crystal X-ray diffraction experiments are, in general, incompatible with spacings expected for the hexagonal defective niccolite phase and any other suggested high-pressure polymorph structure with octahedrally coordinated silicon. In turn, all interplanar distances can be explained by a derivative of the hexagonal close-packed structure with the *c*-axis representing the normal ABAB stacking sequence and an *a*-axis expanded by the factor of √3 compared to an ideal hcp cell (*a* = √3 *a*_hcp_ = √3·2*r*). When taking the crystal ionic radius of oxygen, representative of the size of oxygen atoms in crystal structures^34^ (*r*(O^2−^) = 1.22 Å), the 4.24 Å reflections can be assigned to the ABAB stacking sequence and thus the length of the *c*-axis of the unit cell (*c* = 4*r*·√(2/3)). The 3.71 Å reflections may then be interpreted as the (10$\bar{1}$0) spacing and the 2.71 Å reflections as the (10$\bar{1}$1) spacing. Taking all reflections of the 0001 oriented diffraction pattern, we derive a unit cell with *a* = 4.30(7) Å and *c* = 4.08(7) Å at 26 GPa (Supplementary Table 1). The relatively high errors in the lattice constants of the high-pressure phase may be attributed to the heterogeneous stress field within the single-crystal quartz, resulting from (1) the uniaxial DAC compression and (2) the intrinsic distortion of the sample due to the extreme volume change associated with the high-pressure phase transformation (see Supplementary Information).

The afore-described indexing of reflections implies that the space group symmetry of our high-pressure polymorph must be reduced compared to the hcp base structure because the (0001) and (10$\bar{1}$1) would be absent in a perfect hcp lattice due to the presence of the 6_3_ screw axis and the *c* glide plane. The general absence of systematic reflections also implies that the Bravais lattice is primitive. Furthermore, we observe that the intensities of the six 10$\bar{1}$0 peaks alternate every 60°, as is expected for right and left prisms of trigonal phases. For MX_2_ compounds, there are only three trigonal subgroups of P6_3_/mmc fulfilling these symmetry requirements (P$\bar{3}$1m, P$\bar{3}$m1, and P312), but only for the P$\bar{3}$1m space group (SG = 162) the *a*-axis displays *a* = √3 *a*_hcp_. In fact, the suspected P$\bar{3}$1m space group and the expanded unit cell are adapted by the Li_2_ZrF_6_ structure^35^, which has recently been predicted in a DFT study to form under non-hydrostatic compression^22^. In this structure, the cations are ordered in alternating layers with occupancies of 1/3 and 2/3. It can generally be described as a sheet structure where the layers with an occupancy of 2/3 are made of edge-sharing six-membered Si octahedra parallel to the (0001) plane. These dioctahedral (gibbsite-like) sheets alternate with the layers of isolated Si octahedra that form corner-shared bonds to the dioctahedral sheets above and below the layer (Supplementary Fig. 2). The Li_2_ZrF_6_-type structure is not only isotypic with the aforementioned ɛ-Fe_2_N phase but also with the antimonate mineral rosiaite (PbSb_2_O_4_)^36^. As the latter phase contains oxygen as anion, we propose to consider the metastable high-pressure phase of silica as rosiaite-structured.

### Orientation relationship of rosiaite-structured silica to quartz

In addition to the information on the unit cell, our single-crystal X-ray diffraction patterns provide insight into the geometrical relationship between the quartz host lattice and the rosiaite-structured high-pressure phase. A detailed look at the X-ray diffraction pattern at 26 GPa (Fig. 1b) reveals that the reciprocal directions defined by reflections of the rosiaite phase coincide with those of quartz and some of them (e.g., the 2.7 Å reflection) show a twin-like arrangement. We note also that the indexing of additional reflections is not compatible with a single orientation of the rosiaite phase but rather points to multiple domains with different orientation relationships to the quartz host.

For example, there are six 0001 spots of the rosiaite-structured high-pressure phase at 4.2 Å, which are in the same reciprocal directions as the six 10$\bar{1}$0 spots of the single-crystal quartz. This means that there are three orientations of the high-pressure phase 120° apart. For each of these three orientations, the close-packed (0001) plane is shared with one of the three sets of {10$\bar{1}$0} planes of the host quartz.

**Fig. 5:**
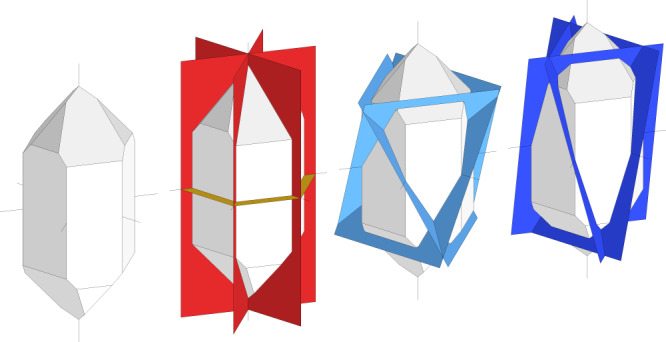
Schematic depiction of the orientation of the (0001) plane of the metastable high-pressure phase with respect to the quartz crystal with its *a* and *c* axes. The orientation of this plane has been inferred from the indexing of diffraction peaks shown in the same colors in Fig. 1c (see also Supplementary Fig. 3). The exact orientation relationships between the rosiaite-structured high-pressure phase (Ros) and quartz (Qtz) are: (0001)_Ros_ // (10$\bar{1}$0)_Qtz_ and (10$\bar{1}$0)_Ros_ // (0001)_Qtz_, (0001)_Ros_ // (0001)_Qtz_ and (10$\bar{1}$0)_Ros_ // (10$\bar{1}$0)_Qtz_, (0001)_Ros_ // (30$\bar{3}$2)_Qtz_ and (11$\bar{2}$0)_Ros_ // (11$\bar{2}$0)_Qtz_, and (0001)_Ros_ // (30$\bar{3}$1)_Qtz_ and (11$\bar{2}$0)_Ros_ // (11$\bar{2}$0)_Qtz_.

In a similar way, the six 10$\bar{1}$0 (3.7 Å) reflections of the high-pressure phase can be assigned to an orientation relationship where the *c* axes of the high-pressure phase and quartz coincide. Furthermore, one can explain the twelve 10$\bar{1}$1 (2.7 Å) and the twelve 10$\bar{1}$2 (1.7 Å) reflections each through three orientations of the high-pressure phase with respect to the host quartz, where the reciprocal <11$\bar{2}$0> directions of both phases are parallel to each other. Altogether, we can assign all reflections of the high-pressure phase to four principle orientation relationships, in which the close-packed (0001) plane of the high-pressure phase is parallel, perpendicular as well as inclined (at 61° and 75°) to the (0001) plane of quartz (Fig. 5, Supplementary Fig. 3).

Beyond the crystallographic orientation relationships, our evaluation of the X-ray diffraction patterns yields no direct information on the morphology and geometry of intergrowth of the rosiaite-structured high-pressure phase with the quartz. It is however noteworthy that the number of orientation relationships between quartz and the high-pressure phase is basically equal to the number of major orientations of amorphous lamellae in the recovered quartz sample (Fig. 4). This supports our previous conclusion that crystalline lamellae of the metastable rosiaite-structured phase may have formed within quartz during compression, which then broke down to glass lamellae during decompression.

## Discussion

To understand the structural response of quartz to dynamic non-hydrostatic loading, we performed rapid membrane-driven DAC experiments on oriented single-crystal quartz with compression rates of 0.5 GPa/s and simultaneously measured time-resolved in situ synchrotron X-ray diffraction patterns. The benefit of these experiments is twofold: (1) the sequence of transformations within quartz could be temporally resolved in the course of the experiments and (2) the single-crystal diffraction patterns yield important information on the symmetry and structure of the phases formed and their orientation relationship to the host quartz.

In detail, our experiments provide evidence for the formation of a metastable high-pressure silica polymorph at pressures above 15 GPa. Based on the symmetry and lattice dimensions, we propose that the phase possesses the rosiaite (PbSb_2_O_6_) structure^36^, which is isotypic to the ɛ-Fe_2_N and the recently proposed Li_2_ZrF_6_ phases but contains exclusively oxygen as anion. Previous studies report in a similar pressure range the occurrence of the so-called quartz II phase, whose structure is unknown to date^15,17,18,27^. Although the important interplanar spacing of 4.2 Å (*c*-axis for rosiaite) is not reported in these studies, we speculate that the rosiaite-structured phase could be indeed quartz II.

Furthermore, our X-ray diffraction patterns and TEM observations indicate that the rosiaite phase forms during compression as lamellae in various crystallographic relationships to quartz. Since the phase is metastable, it collapses to the amorphous state during decompression. The metastability of the rosiaite phase might be related to the repulsive forces exerted by silicon cations in the dioctahedral layers of its sheet structure which might limit its stability according to Pauling’s third rule^37^. In these layers, SiO_6_ octahedra have three edges shared with neighboring octahedra, while in the stable high-polymorphs stishovite and seifertite, there are only two edges shared.

In terms of their appearance, thickness, and orientation, the amorphous lamellae produced in our rapid DAC compression experiments resemble closely PDFs in shocked quartz^5,30,38,39^. They are sharp lamellar discontinuities with thicknesses at the nanometer scale and are parallel to the basal (0001), rhombohedral {10$\bar{1}$1}, and prismatic {10$\bar{1}$0} crystallographic planes. This resemblance is remarkable since the strain rates in our experiments (10^−2^ to 10^−3^ s^−1^) are about four orders of magnitudes slower than those of natural impacts. On the other hand, deformation in well-established shock experiments (strain rates: 10^6^ to 10^7^ s^−1^) is distinctly faster and also results in the formation of amorphous lamellae^10,40^ at typical pressures of 25–30 GPa. Thus, quartz apparently reacts in a similar way over a wide range of strain rates, which might be explained by the generally sluggish transformation kinetics of silica to stable high-pressure polymorphs. On the contrary, a recent DFT study suggests that the metastable rosiaite phase forms readily under non-hydrostatic conditions by a displacive mechanism from quartz, which could thus instantaneously occur even under very high strain rates^22^. From these considerations, we infer that impact-produced PDFs may be regarded as the amorphization product of the lamellar-shaped metastable rosiaite phase.

**Fig. 6:**
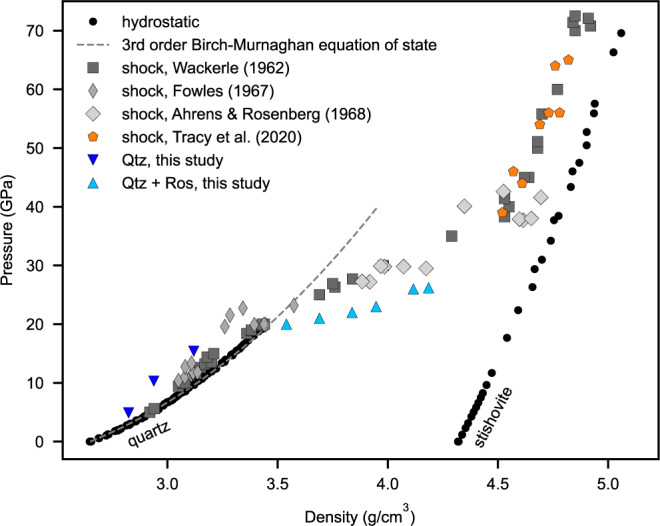
Pressure-density diagram displaying Hugoniot^12,42, 43^ and X-ray diffraction data^19^ derived from shock experiments on quartz in comparison to density data of hydrostatic diamond anvil cell (DAC) experiments^55, 56^ and to our rapid compressed samples, which represent a mixture of quartz (Qtz) and the metastable rosiaite-structured (Ros) high-pressure phase during compression. The densities of the individual phases have been inferred from measured lattice parameters and the proportions of the two phases were derived from Fig. 2.

The close resemblance of our experimentally produced amorphous lamellae to PDFs in shocked quartz may furthermore indicate the relevance of our data for the interpretation of the Hugoniot curve of quartz in that the mixed phase region of the Hugoniot curve could basically reflect a mixture of quartz and the metastable rosiaite high-pressure polymorph. In general, our observation of amorphization occurring only during decompression disproves earlier ideas that a dense glass forms directly during compression^10,41^. According to our results, it is also unlikely that the stable high-pressure polymorph stishovite is the phase that accounts for the sudden density change in the mixed phase region. The only in situ shock study on quartz arrives at the same conclusions but reports the formation of the defective niccolite phase. It has however to be noted that the two types of rapid compression experiments, membrane-driven DAC versus gas gun, were performed at different pressures, temperatures, and timescales. As the crystal structures and densities of both phases, defective niccolite (4.63 g/cm^3^ at 42 GPa) and rosiaite-structured silica (4.59 g/cm^3^ at 26 GPa), are very similar, an increasing amount of any of the two high-pressure phases in the mixed phase region could readily explain the abrupt jump in specific volume^12,13,19^. Here, we have quantified the phase fractions during compression and thus we can demonstrate this for rosiaite-structured silica by plotting the bulk densities of our samples for various pressures in comparison to shock Hugoniot data of single-crystal quartz^12,42–45^ and X-ray diffraction results from the recent in situ gas gun study^19^ (Fig. 6). Overall, our mDAC data match the progression of the pressure-density Hugoniot curve in the low-pressure range from 5 to 26 GPa. The sudden density increase, which marks the onset of the mixed phase regime, is also at about 20 GPa but the densities of our DAC samples in this regime (20–26 GPa) seem to be slightly higher than those of shock-compressed quartz at given pressures. The reason for this deviation could be indeed the difference in temperatures. Our quartz samples are compressed at room temperatures, while the temperatures of shock-compressed quartz in the mixed phase region are commonly on the order of 300–500 °C^10,12,40^. The shock gas gun data match well the Hugoniot curve at high pressures between 40 and 65 GPa, where the shock temperatures are also significantly enhanced and the phase transformation should be completed^19^. Without further experiments exploiting comprehensively the influences of pressures, temperatures, and strain rates on the phase transformations, it seems difficult to conclusively interpret the Hugoniot curve from the onset of the mixed phase region to the very high-pressure regime. As the metastable rosiaite and defective niccolite structures basically differ with respect to the degree of silicon ordering at the octahedral sites, there could be, for example, a continuous transition between the two phases, which would be influenced by the varying temperatures along the Hugoniot curve.

Finally, our observation of a metastable high-pressure polymorph of silica may generally shed more light on the phenomenon of pressure-induced amorphization observed also in various other material systems (e.g., ice^46,47^), which sometimes even do not develop glass by rapid quenching of melt^46^. We speculate that the formation of metastable crystalline phases could be one pathway for pressure-induced amorphization of rapidly compressed materials at room temperature. If the compression occurs, however, at a certain elevated temperature, the metastable high-pressure silica phase may be regarded as a precursor structure of stable high-pressure polymorphs, in which diffusion of the silicon atoms into an ordered atomic configuration may be facilitated. Which of the stable high-pressure phases is formed upon diffusion ultimately depends on the timing, heat, and pressures introduced during the compression process and this might unify the seemingly contradictory results concerning the formation pressures and stability fields of high-pressure polymorphs under impact conditions.

## Methods

### Realization of compression experiments

For the purpose of mDAC experiments, we have prepared thin sections (thickness ~30 µm) from single crystalline natural quartz and coated them with a thin layer of gold (thickness ~100 nm) as an internal pressure standard. Disc-shaped samples (diameter 130 µm) were produced from these thin sections using focused ion beam (FIB) preparation^48,49^ and loaded into double membrane-driven diamond anvil cells (mDACs)^50^ without a pressure transmitting medium.

The experiments with uniaxial compression and simultaneous single-crystal X-ray diffraction were carried out at the Extreme Conditions Beamline (ECB) P02.2^51^ at the third-generation light source PETRA III, DESY, Germany, using a monochromatic X-ray beam with the energy of 25.6 keV (wavelength of 0.4843 Å). The X-ray beam was focused on an area of 8 × 3 μm^2^ with a compound refractive lens system. X-ray diffraction images were collected on a Perkin Elmer area detector (PE XRD1621). The sample-detector distance and tilt of the detector were calibrated using a CeO_2_ standard (NIST 674b). The conversion of the diffraction images to one-dimensional diffractograms was carried out with the software DIOPTAS^52^.

At the beginning of an experiment, the pressure was increased carefully in small steps until signs of a shift or broadening of the diffraction peaks indicated the start of compression. Immediately thereafter, samples were compressed to the peak pressure of 26 GPa employing an average compression rate of 0.5 GPa/s. The peak pressure was held constant for approximately 2 min, followed by complete decompression with the average rate of 0.3 GPa/s. During the entire compression-decompression cycle, diffraction images were collected with an acquisition time of 2 s (Supplementary Fig. 1).

### Transmission electron microscopy

For the investigations at the TEM, the recovered single-crystal samples were prepared by the FIB technique using a FEI Quanta 3D FEG workstation at the Friedrich Schiller University Jena^49^. In the sample center, a section was cut perpendicular to the surface with a gallium ion gun operated at an acceleration voltage of 30 kV and a beam current between 0.1 and 30 nA. The section was thinned and finally cleaned using the acceleration voltage of 5 kV at the beam current of 48 pA, resulting in a final thin section with a size of approximately 20 × 10 μm^2^ and a thickness between 100 and 200 nm. Immediately after FIB preparation, the section was investigated using the FEI Tecnai G2 FEG TEM operated with an acceleration voltage of 200 kV.

### Evaluation of X-ray diffractograms

The evaluation of the X-ray diffractograms involved first the removal of the high background, which originates from the Compton scattering of the diamonds. The background was determined by a least squares polynomial fit in each diffractogram using reference points that were set manually at local minima. Care was taken to use the same positions of the reference points for a wide range of diffractograms, some adjustment was however necessary due to the appearance/disappearance and shift of diffraction peaks. Twelfth-order polynomial functions were then fit to the reference points. The fitted functions were finally subtracted from the measured diffractograms to obtain the background-free diffractograms.

Using the software package LMFIT^53^, peak positions and integrated intensities were obtained from single peak fits assuming a Voigt distribution function for the peak shape. Pressures were calculated from the positions of the 111, 200, 220, 311, and 222 diffraction peaks of gold employing the third-order Birch–Murnaghan equation of state parameterized in ref. ^54^.

The intensities of the quartz peaks vary strongly at the beginning of the experiments and peaks broaden distinctly due to the heterogeneous stress distribution developing during incipient compression of the sample. Therefore, we scaled the intensity-time curve of the 10$\bar{1}$1 peak of quartz to a value of 1, when the intensity variations stopped, i.e., before the transformation to rosiaite-structured silica (Fig. 2). The normalized intensity of the 10$\bar{1}$1 peak of quartz thus provides a measure of the phase fraction of quartz in the course of the experiment. Similarly, the intensity curve of the high-pressure phase and the glass were scaled with multiplication factors derived from images with the maximum peak and diffuse background intensities, respectively. The scaled intensities of all phases coexisting at a certain time during the experiments were finally normalized to a value of 1 to provide information on the phase fractions at every point during the experiment.

## Supplementary information

Supplementary Information file

## Data Availability

Datasets for Figs. 2, 3, and 6 can be found in the Supplementary Data. Source data are provided with this paper.

## Source data

Source Data

## Code availability

The computer code used to generate the results reported in this study is available from the corresponding author upon request.
