# Supplementary material for: Evidence for a rosiaite-structured high-pressure silica phase and its relation to lamellar amorphization in quartz
Source: Nat Commun. 2023 Feb 4;14:606. doi: 10.1038/s41467-023-36320-7 (PMC9899207; doi:10.1038/s41467-023-36320-7)
Supplement: Supplementary file 1 — Supplementary Information file [file 41467_2023_36320_MOESM1_ESM.pdf]

Supplementary Information

Evidence for a rosiaite-structured high-pressure silica phase and its relation to lamellar amorphization in quartz

Christoph Otzen[1,2], Hanns-Peter Liermann[2], Falko Langenhorst[1,3]*

[1]Institute of Geoscience, Friedrich-Schiller-University Jena, Carl-Zeiss-Promenade 10, 07745 Jena, Germany

[2]Deutsches Elektronen-Synchrotron DESY, Notkestr. 85, 22607 Hamburg, Germany,

[3]Hawai'i Institute of Geophysics and Planetology, School of Ocean and Earth Science and Technology, University of Hawai'i at Manoa, Honolulu, HI 96822, USA.

*Corresponding author. Email: Falko.Langenhorst@uni-jena.de

Integrated, background-free diffractograms

Selected background-free diffractograms were obtained by integration of the two-dimensional diffraction images and subtraction of the fitted background (Supplementary Fig. 1). Base points used for the determination of the background in each raw diffractogram, as described in the methods section, are also displayed. In support of the results described in the main text, the diffractograms describe the crystallographic changes of the quartz sample in the course of the experiment. The four strongest diffraction peaks of the rosiaite-structured phase, namely the 0001, $10\bar{1}0$, $10\bar{1}1$ and $10\bar{1}2$ peaks appear in the diffractograms at pressures above 15 GPa. The other peaks attributed to the rosiaite-structured phase appear within the same pressure range, but their intensities are low and thus cannot be seen in the diffractograms.

Indexing and interplanar spacings of the diffraction peaks of rosiaite-structured SiO_2

The total number of observed diffraction peaks attributed to the rosiaite-structured phase are listed in Supplementary Table 1 with their Miller indices and interplanar spacings. Using the software UnitCell[1], parameters of the trigonal unit cell were calculated from the measured interplanar spacings, resulting in a = 4.30(7) Å and c = 4.08(7) Å, from which the calculated interplanar spacings were then determined. The majority of measured and calculated interplanar spacings agree well, whereas larger deviations can be seen for the 0001 and $10\bar{1}2$ reflections. It has to be noted that the reflections are from four differently oriented lamellae and that we uniaxially

compress samples, which may explain, in part, the deviations in measured and calculated interplanar spacings. We attribute these deviations, however, also to the heterogeneous stress field within the sample resulting from the high-pressure phase transformation of quartz to rosiaite-structured silica. When rosiaite-structured lamellae form within quartz, the sample must locally accommodate the huge density difference between rosiaite-structured silica (ρ=4.59 g/cm^3) and quartz (ρ=2.65 g/cm^3). The sudden volume decrease (~70 % with respect to quartz) may result in a strong distortion of samples and the observed deviations in interplanar spacings.

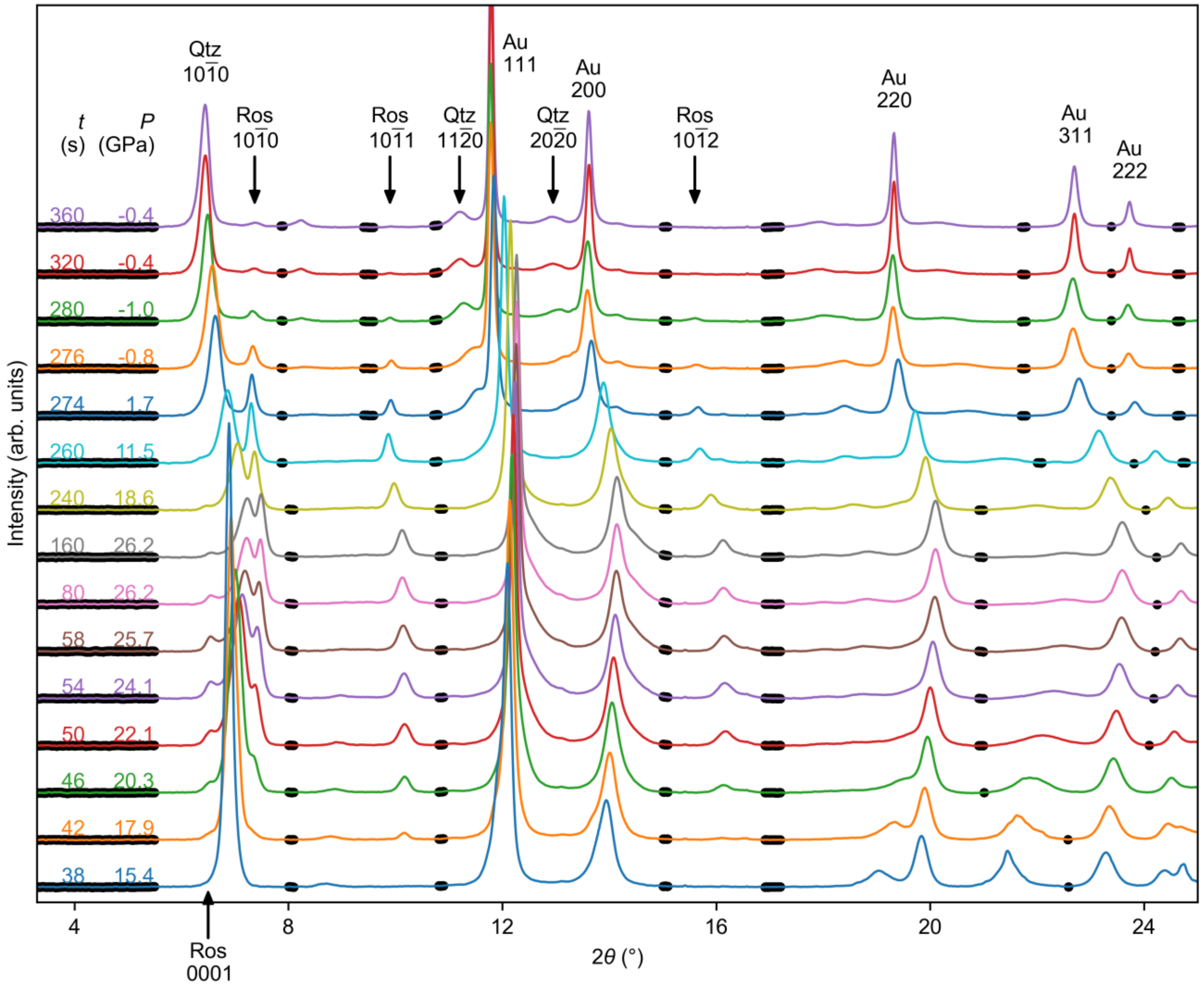

Supplementary Fig. 1. Selected X-ray diffraction profiles. They are derived by integration of diffraction patterns of single crystal quartz and polycrystalline gold standard. The profiles depict the shift of diffraction peaks of quartz during the compression and decompression and clearly show the appearance of the metastable high-pressure phase reflections, starting at about 15 GPa. The black symbols mark the fixed reference positions used for the polynomial background fit.

Supplementary Table 1. Observed and calculated interplanar spacings *d* of the rosiaite-structured SiO_2 phase. The calculated values were calculated using the refined cell parameters *a* = 4.30 (7) Å and *c* = 4.08 (7) Å.

hkil	*d* (Å), observed	*d* (Å), calculated
0001	4.24	4.08
$10\bar{1}0$	3.71	3.72
$10\bar{1}1$	2.742	2.75
$11\bar{2}0$	2.16	2.15
0002	2.12	2.04
$11\bar{2}1$	1.925	1.90
$10\bar{1}2$	1.725	1.79
$20\bar{2}1$	1.695	1.69

Crystal structure of the rosiaite-structured SiO_2 phase

The crystal structure of the rosiaite-structured SiO_2 phase, determined from the intensities and positions of X-ray diffraction peaks described in the main text, is shown in Supplementary Fig. 2. The structure can be regarded as a hcp arrangement of oxygen atoms located at the corners of the coordination octahedra. In the ABAB stacking sequence along the *c* axis, the available octahedral interstices of the intermediate layers are alternatingly occupied by 1/3 and 2/3 with silicon atoms. Within the layers with occupancy 1/3, the resulting coordination octahedra are isolated from each other, which are depicted blue in Supplementary Fig. 2. In the layers with occupancy 2/3, namely the dioctahedral sheets, each of the coordination octahedra shares edges with three neighboring octahedra, which are depicted orange in Supplementary Fig. 2. The layers/sheets are interconnected via the corners of the isolated (blue) octahedra.

Single crystal diffraction images and crystallographic orientation relationships

In addition to the descriptions and interpretations of the diffraction images (Fig. 1) in the main text, it should be pointed out that the collected diffraction patterns are very similar to single crystal X-ray precession images (i.e. two-dimensional sections through reciprocal space) expected for the respective orientations. Rotations were not employed in the present experiments, therefore, only few diffraction peaks are expected to appear at low diffraction angles according to the Ewald reconstruction of the experiments. We attribute the appearance of additional reflections at higher

diffraction angles to the increasing strain that builds up in the single crystal discs during uniaxial compression. This peculiar effect facilitated the interpretation of diffraction patterns to explain the crystallographic orientation relationships between quartz and the rosiaite-structured phase. These are derived from the X-ray diffraction pattern in Fig. 1c and are depicted by means of the orientation of the (0001) plane of the rosiaite-structured phase with respect to a quartz crystal in Fig. 5.

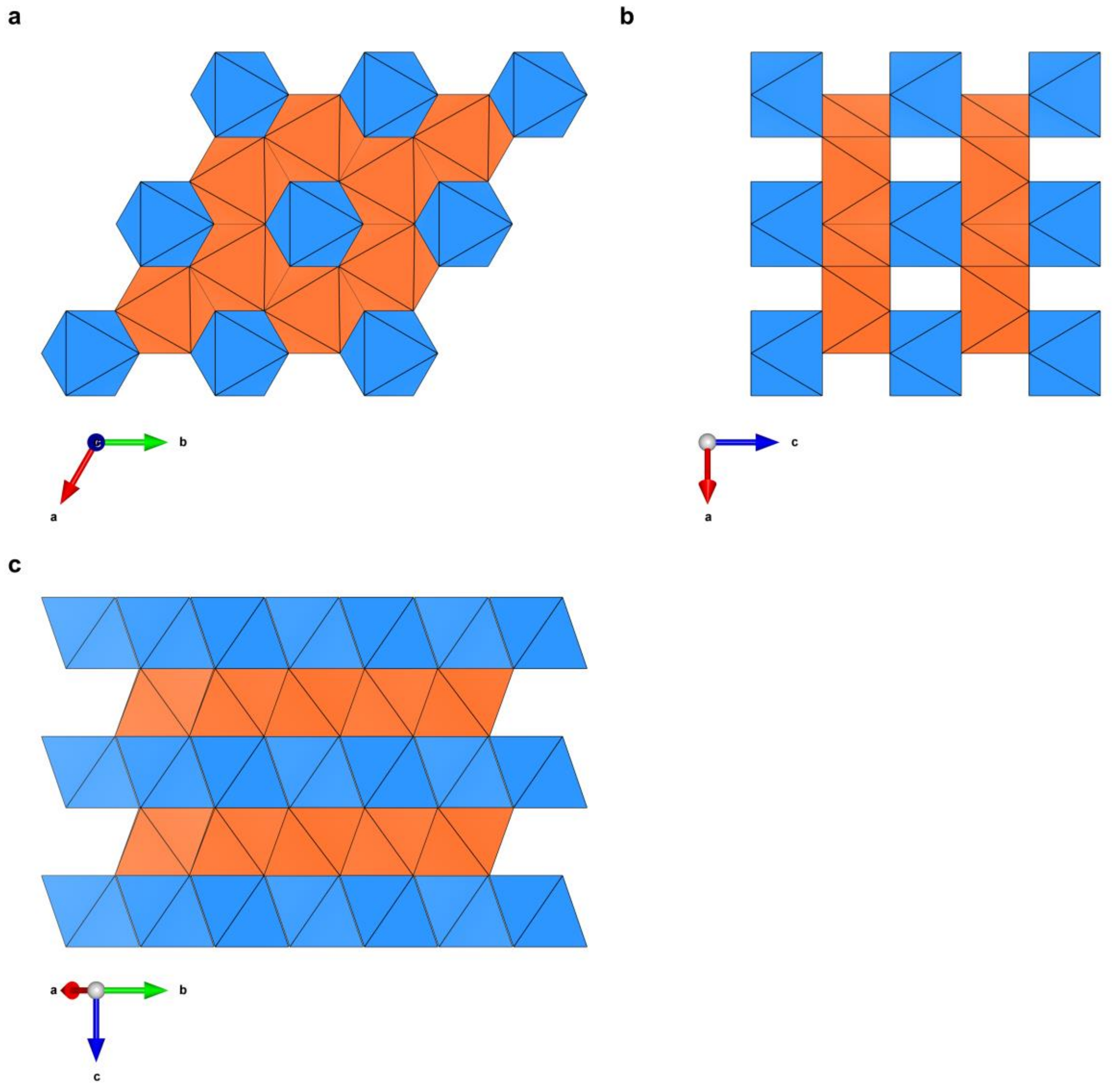

Supplementary Fig. 2. Crystal structure of the rosiaite-structured SiO_2 phase. The projections of the crystal structure correspond to three orthogonal directions along (a) [001], (b) [010] and (c) [210]. In this sheet structure, sheets of isolated SiO_6-octahedra (blue) and sheets of three-fold edge-shared SiO_6-octahedra (orange) alternate along the *c* axis.

For the readers convenience, we show additionally a schematic illustration of the orientation relationships in Supplementary Fig. 3. The figure indicates hypothetical diffraction images of single domains of each crystallographic orientation relationship. The remaining single crystal-like diffraction peaks of the two other domains are obtained by rotation of the indicated domains by 120° and 240° around the primary beam. Subsequent merging of all four orientations produces the complete diffraction image shown in Fig 1c.

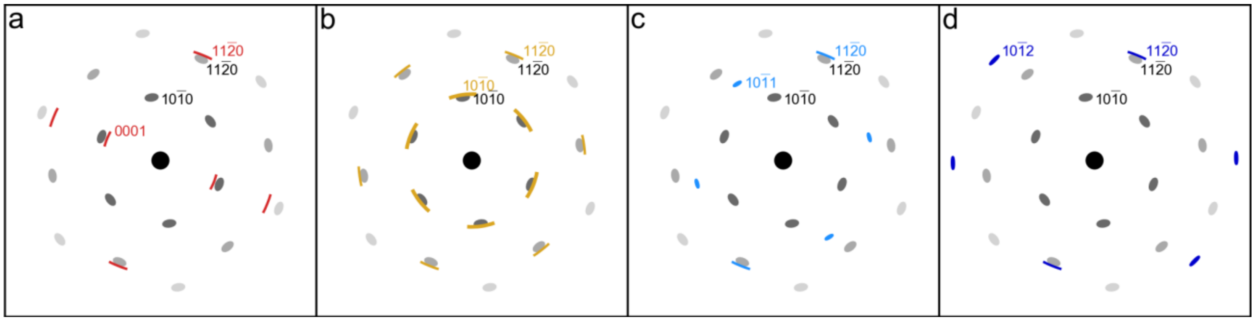

Supplementary Fig. 3. Schematic X-ray diffraction images of quartz viewed along the *c* axis and single domains of the rosiaite-structured phase. The viewing directions of the domains are along the zone axes (a) [$\bar{1}$10], (b) [001], (c) [$\bar{1}$11] and (d) [$\bar{2}$21], corresponding to the single orientations with respect to quartz illustrated in Fig. 5.

Supplementary references

1. Holland, T. J. B. & Redfern, S. A. T. Unit cell refinement from powder diffraction data: the use of regression diagnostics. *Miner. Mag.* **61**, 65–77 (1997).
